# Elucidation of the Interaction between Flavan-3-ols and Bovine Serum Albumin and Its Effect on Their In-Vitro Cytotoxicity

**DOI:** 10.3390/molecules24203667

**Published:** 2019-10-11

**Authors:** Yasuyuki Fujii, Yoshitomo Suhara, Yusuke Sukikara, Tomohiro Teshima, Yoshihisa Hirota, Kenjiro Yoshimura, Naomi Osakabe

**Affiliations:** 1 Department of Bio-Science and Engineering, Shibaura Institute of Technology, 307 Fukasaku, Munumaku, Saitama 337-8570, Japan; mf17059@shibaura-it.ac.jp (Y.F.); suhara@sic.shibaura-it.ac.jp (Y.S.); bn14036@shibaura-it.ac.jp (Y.S.); BN15037@shibaura-it.ac.jp (T.T.); 2 Department of Machinery and Control Systems, Shibaura Institute of Technology, 307 Fukasaku, Munumaku, Saitama 337-8570, Japan; hirotay@sic.shibaura-it.ac.jp (Y.H.); kenjiroy@sic.shibaura-it.ac.jp (K.Y.)

**Keywords:** Flavan-3-ols, bovine serum albumin, docking simulation, B-type procyanidin, cytotoxicity

## Abstract

Flavan-3-ols (FLs), specifically catechin and its oligomer B-type procyanidins, are suggested to potently bind to bovine serum albumin (BSA). We examined the interaction between BSA and FLs by fluorescence quenching and found the following order of binding activities to BSA: cinnamtannin A2 (A2; tetramer) > procyanidin C1 (C1; trimer) ≈ procyanidin B2 (B2, dimer) > (−)epicatechin (EC, monomer). Docking simulations between BSA and each compound at the binding site showed that the calculated binding energies were consistent with the results of our experimental assay. FLs exerted cytotoxicity at 1000 μg/mL in F11 cell culture with fetal bovine serum containing BSA. In culture containing serum-free medium, FLs exhibited significant cell proliferation at 10^−4^ μg/mL and cytotoxicity was observed at concentrations greater than 10 μg/mL. Results of this study suggest that interactions between polyphenols and BSA should be taken into account when evaluating procyanidin in an in vitro cell culture system.

## 1. Introduction

Flavan-3-ols (FLs) are a subclass of plant flavonoids possessing a diphenylpropane structure, and are categorized as monomeric or oligomeric catechins [1]. These compounds are present as astringent substances in chocolate [2], red wine [3], black soy beans [4] and some other foods as well. In particular, B-type procyanidins, which are catechin oligomers linked by C4–C8 bonds, are known to be rich in these types of foods. Foods rich in B-type procyanidins may exhibit significant potential for management of cardiovascular health [5,6].

Numerous randomized controlled trials have confirmed that foods rich in FLs are associated with beneficial effects in several conditions that contribute to metabolic syndrome, including hypertension [7], dyslipidemia [8], and glucose intolerance [9]. To elucidate these beneficial mechanisms of FLs, a number of in vitro studies have been carried out on vascular endothelial function [10,11], improvement of lipid metabolism [12,13] and glucose intolerance [14,15]. All of these in vitro studies were carried out using cell culture with bovine serum albumin (BSA) containing fetal bovine serum (FBS).

In contrast, FLs, including B-type procyanidin, are known to exhibit high affinities to proteins [16,17]. B-type procyanidin oligomers have also been reported to strongly quench the intrinsic fluorescence of BSA through a static quenching procedure, while similar results were not observed with the monomers [18]. These results suggest that affinity with proteins may differ depending on the degree of polymerization of B-type procyanidin. Moreover, it was suggested that the interaction of procyanidin and BSA might affect in vitro cell experiments using an FBS-supplemented medium containing BSA.

In the present study, we investigated the binding affinity of FLs and B-type procyanidins, namely (−)-epicatechin (EC), procyanidin B2 (B2), procyanidin C1 (C1), and cinnamtannin A2 (A2), to BSA by the fluorescence quenching method together with docking simulations. In addition, we examined the influence of the interaction of FLs and BSA on the cytotoxicity of FLs in an in vitro cell culture, and also compared the results to the cytotoxicity of B-type procyanidins.

## 2. Results

### 2.1. Interaction between FLs and BSA

**Figure 1:**
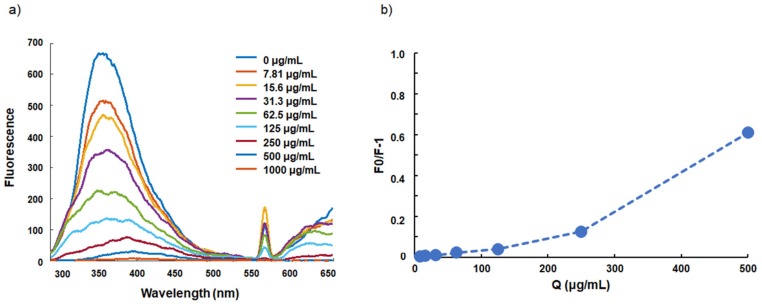
Emission spectra of bovine serum albumin (BSA) in the presence of different concentrations of Flavan-3-ols (FLs) (**a**), and Stern–Volmer plot (**b**), where F and F_0_ are the emission intensities in the presence and absence of FLs, respectively, measured at the emission wavelength of 350 nm.

The emission spectra of BSA without FLs and at different concentrations of FLs are shown in Figure 1a. At λ_ex_ = 280 nm, BSA showed an intense emission band centered at ~350 nm. With subsequent increases in the concentration of FLs (to concentrations between 7.81–1000 µg/mL) the intensity decreased. In addition, a narrow emission band with its peak centered at 563 nm was also detected upon addition of FLs. The intensity of this band decreased with increasing concentration of FLs; the Stern–Volmer plot is shown in Figure 1b.

### 2.2. Interaction between BSA and (−)-Epicatechin and Procyanidins

**Figure 2:**
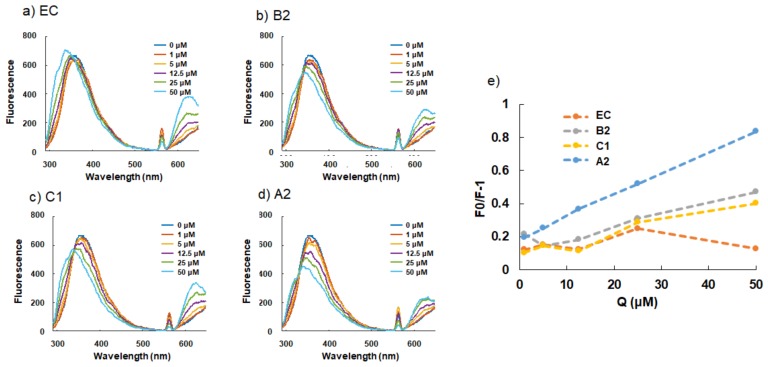
Emission spectra of BSA in the presence of different concentrations of EC (**a**), B2 (**b**), C1 (**c**), A2 (**d**) and Stern–Volmer plots (**e**). F and F_0_ are the emission intensities in the presence and absence of EC, B2, C1 or A2, respectively, measured at the emission wavelength of 350 nm.

The fluorescence spectra of BSA without procyanidins and at different concentrations of EC, B2, C1, and A2 are shown in Figure 2a–d. Upon addition of A2, the fluorescence intensity of the emission band at ~350 nm was decreased. With subsequent increases in the concentration of A2 (from 1–50 μM), the intensity further decreased. These changes were also observed upon addition of B2 or C1, but not upon addition of EC. In addition to the emission band centered at ~350 nm, a narrow emission band with its peak centered at 563 nm was detected upon addition of procyanidins at different concentrations, and this peak was decreased with subsequent increases in the concentration of each. The emission peak shifted to lower wavelengths with subsequent increases in the concentrations of these compounds.

The Stern–Volmer plots of EC, B2, C1 and A2 are shown in Figure 2e. A2 was found to highly interact with BSA, while B2 and C1 were found to slightly interact, and EC demonstrated hardly any interaction with BSA.

The quenching constants (Kq) for the interaction of EC, B2, C1 and A2 with BSA, which were calculated by the Stern–Volmer equation, are shown in Table 1. A2 showed the highest Kq value among the procyanidins. B2 and C1 exhibited nearly the same Kq, while that of EC was very low.

### 2.3. Docking Studies

**Figure 3:**
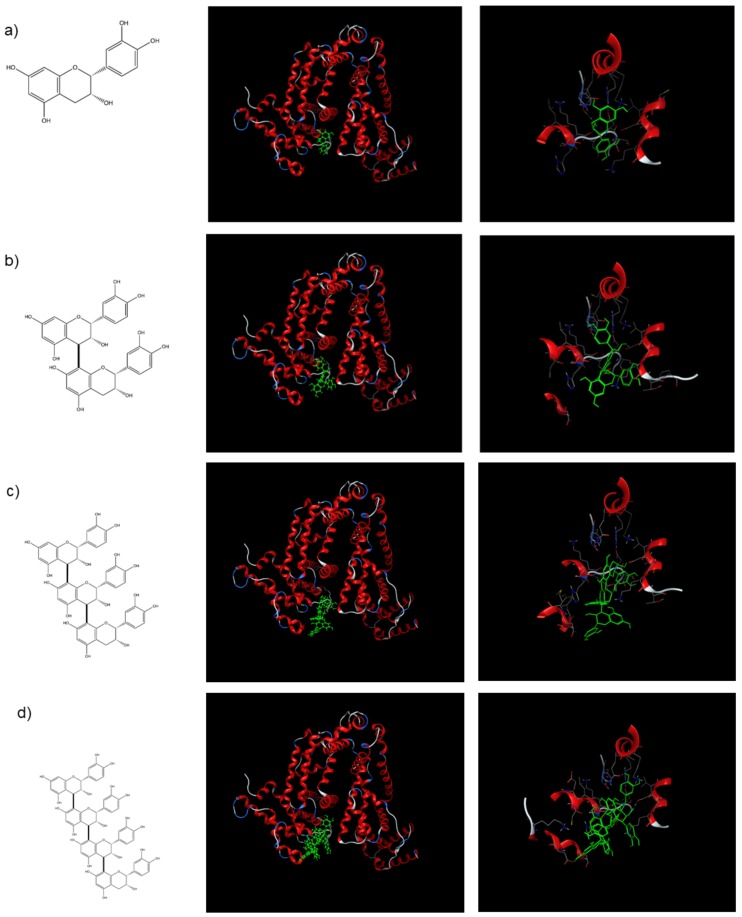
Chemical structure and lowest-energy docked poses of (−)-epicatechin (**a**), procyanidin B2 (**b**), procyanidin C1 (**c**), and cinnamtannin A2 (**d**) with BSA. Docked poses reveal the entire ribbon structure of the protein shown in the left panel. The right panel shows close-ups of the docked poses.

To better understand the binding modes of FLs to BSA at the atomic level (based on the results shown in Figure 2), we performed molecular docking studies on FLs with BSA (PDB ID: 4JK4) using the docking program of the MOE suite (see Computational Details) as shown in Figure 3. We calculated the binding affinity by replacing 3,5-diiodosalicylic acid, originally bound to BSA, with FLs using the “dock” mode. Results suggest that the binding energy decreased as the unit became longer (EC < B2 < C1 <A2). This indicates that the order of binding affinities was: EC < B2 < C1 <A2, which was different from our experimental assay results of: EC < C1 < B2 <A2, as shown in Figure 2. As we hypothesized that the binding between FLs and BSA is relatively non-specific, we then searched other possible binding sites of BSA and calculated the corresponding binding energies. The lowest-energy docked poses of FLs with BSA are shown in Figure 3. The docking results positioned FLs within the BSA binding sites, and the FLs (EC, B2, C1, and A2) with the lowest binding energy displayed affinities of −6.36 kcal/mol, −7.57 kcal/mol, −7.39 kcal/mol, and −8.63 kcal/mol, respectively. Half of the C1 molecule bound to the binding site of BSA, while the whole B2 molecule bound to the same binding site. We predicted that this is the reason why the binding energy of C1 was lower than that of B2. This result suggests that the binding affinities of the FLs to BSA are on the following order: EC < B2 ≃ C1 < A2, which is consistent with the assay results shown in Figure 2.

### 2.4. Influence of BSA on Cytotoxicity of FLs

**Figure 4:**
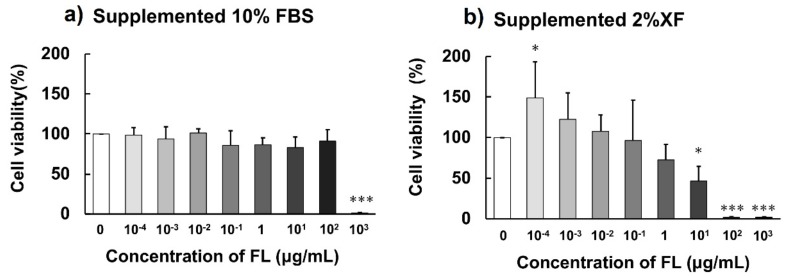
F10 cell viability after 48-h incubation in the presence of different concentrations of FLs in control culture medium supplemented with 10% fetal bovine serum (FBS) (**a**) or 2% NutriStem V9 XF Basal Medium (XF) (**b**). Each value represents mean ± SD (*n* = 5). Significant differences vs. no addition (0 μg/mL): * *p* < 0.05, *** *p* < 0.001.

Cell viability following incubation of FLs with 10% fetal bovine serum (FBS) or with 2% XF (NutriStem V9 XF Basal Medium, Biological Industries Ltd., Kibbutz Beit Haemek, Israel) is shown in Figure 4. For F11 cells cultured in a medium supplemented with 10% FBS, FLs did not exert any cytotoxicity up to a concentration of 100 μg/mL; however, most cells died at a concentration of 1000 μg/mL. In contrast, when cultured in serum-free medium (with 2% XF), significant cell proliferation was observed at a concentration of 10^−4^ μg/mL, while 50% cell death was observed at a concentration of 10 μg/mL, and nearly 100% cell death was observed at concentrations of 100 and 1000 μg/mL.

### 2.5. Comparison of Cytotoxicity of Procyanidins

**Figure 5:**
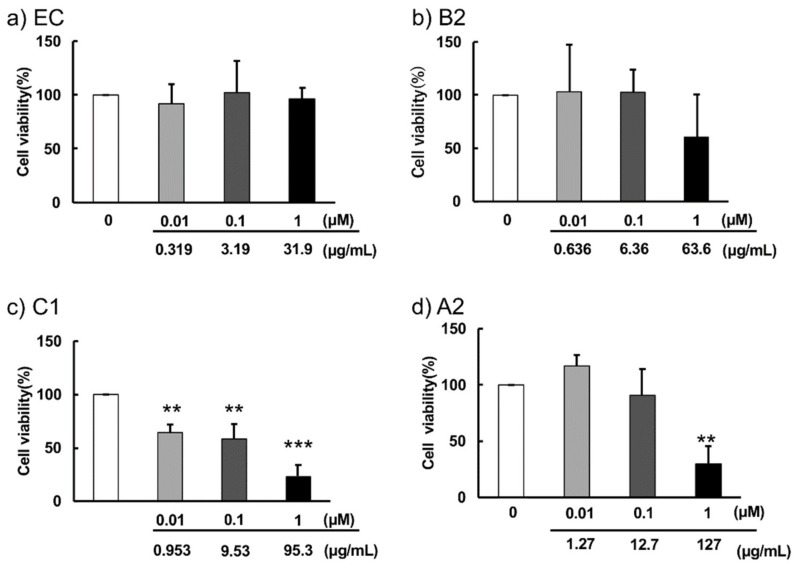
F10 cell viability 48 h after incubation in the presence of different concentrations of EC (**a**), B2 (**b**), C1 (**c**) or A2 (**d**) in control culture medium supplemented with 2% NutriStem V9 XF Basal Medium (XF). Each value represents mean ± SD (*n* = 5). Significant differences vs. no addition (0 μg/mL): ** *p* < 0.01, *** *p* < 0.001.

Cell viability after 48-h incubation with EC, B2, C1 or A2 with 2% XF is shown in Figure 5.

EC did not result in any toxicity at concentrations of 0.01 to 1 μM, and B2 showed slight cytotoxicity at 1 μM. Upon addition of C1, ~30%, ~40%, and ~70% cell death was observed at concentrations of 0.01 μM, 0.1 μM, and 1 μM, respectively. In contrast, ~70% of cell death was observed at a concentration of 1 μM upon addition of A2, while cytotoxicity was not observed at concentrations lower than 1 μM.

## 3. Discussion

BSA contains two tryptophan residues (Trp 134 and 212) that possess intrinsic domains, and each domain, in turn, produces fluorescence (λ_EX_ = 280 nm, λ_EM_ = 350 nm, as shown in Figure S1) [19]. BSA is widely used as a model protein to study the binding interactions of small molecule compounds with proteins.

FLs were found to exhibit dose-dependent binding affinity to BSA (Figure 1). The emission band centered at ~350 nm was decreased with increasing concentrations of FLs; moreover, a narrow emission band centered at 563 nm appeared (Figure 1a). These changes were reported in a previous study of resveratrol (a type of polyphenol), as well as FLs [20]. Polyphenols are a group of compounds that have several hydroxyl groups, similar to catechol, pyrogallol, etc. It is well known that catechol easily undergoes oxidation in a pH-dependent manner. In this process, reactive oxygen species (ROS) such as superoxide and hydrogen peroxide are produced, and subsequent ROS result in the formation of intermediate compounds as well [21]. These intermediate compounds are unstable, and thus it is difficult to identify them. Whether this sharp peak is derived from the bond between BSA and that of polyphenol, FLs or resveratrol, remains unclear, and thus further investigation is needed.

Among the various oligomers of B-type procyanidin, the tetramer A2 had a high affinity for BSA, while the dimer (B2) and trimer (C1) had slight effects (Figure 2). The low binding affinity of EC to BSA is consistent with previous reports [18]. The calculated Kq values were also consistent with these results (Table 1). In addition, the emission peak at ~350 nm was slightly shifted toward a lower wavelength with subsequent increases in the concentrations of EC, B2, and C1 or A2 (Figure 2a). The maximum fluorescence intensity of these compounds was shown to be ~320 nm (Figure S1), which may have been due to some interference with the measurement.

It has been reported that the fluorescence quenching of BSA can be divided into three quenching mechanisms, namely static quenching caused by formation of a ground–state complex of protein with quenchers, dynamic quenching caused by the collision of protein and quenchers, and combined dynamic and static quenching caused by both collision and complex formation with the same quencher, respectively [22,23]. As shown in Figure 1b and Figure 2e, plots of F_0_/F vs. [Q] were nonlinear in the low concentration range of FLs and procyanidins, indicating that the induced quenching mechanism of BSA may be via combined dynamic and static quenching.

In the present study, we confirmed that the interaction between BSA and FLs exerted a significant influence on the cytotoxicity of FLs (Figure 4). It is known that albumin is a major protein component of FBS used in cell culture experiments. In the presence of FBS, FLs did not exert any cytotoxicity up to concentrations of 100 μg/mL, while most cells died at a concentration of 1000 μg/mL. In contrast, ~50% cell death was observed at FLs concentration of 10 μg/mL, and ~100% cell death was observed at FLs concentrations of 100 and 1000 μg/mL without FBS. These results suggest that at concentrations of 100 μg/mL, most FLs are bound to BSA in FBS and do not exhibit any cytotoxicity. A number of in vitro cell culture experiments have been conducted to elucidate the physiological effects of procyanidins and other polyphenols; however, almost all of these studies were conducted in the presence of FBS.

Among the polyphenols, it has been reported that there are various compounds that exhibit high affinity for BSA, such as the gallate-type catechin [24], resveratrol [20] and procyanidins [16,17]. Results of this study suggest that when evaluating polyphenols in in vitro cell culture experiments, a serum-free medium should be used instead of FBS.

In addition, under serum-free medium conditions, FLs showed cell proliferation activity at low concentrations (10^−4^ μg/mL). It was previously reported that polyphenols exhibit hormetic dose responses in various cell lines. Specifically, low concentrations of polyphenols were shown to enhance cell proliferation, whereas higher concentrations were found to be inhibitory [25]. In addition, it was reported that the hormetic activity of phenolic substances might involve the NF-E2-related factor (Nrf) 2 pathway [26]. The present results suggest that FLs exhibit hormetic effects in the presence of the F11 cell line, which is a N18TGneuroblastoma X rat DRG sensory neuron hybrid cell line.

Cytotoxicity intensities of procyanidins to F11 cells were as follows: C1 (trimer) > A2 (tetramer) >> B2 (dimer) = EC (monomer). This intensity did not correlate with BSA affinity. The number of hydroxyl groups of the procyanidins was also found to not be important for cytotoxicity. B-type procyanidins may have a common core structure that is necessary for interactions with a target biomolecule. Polymerized 2-phenyl-3,4-dihydro-2H-chromen-3-ol is a possible core structure (Figure 3); In particular, C1 has been suggested to exhibit a complementary relationship with the target molecule to induce cytotoxicity. Further studies are needed to elucidate the interactions between the target biomolecule and B-type procyanidin.

In conclusion, the results of the present study suggest that the interaction of polyphenols and BSA should be taken into account when evaluating polyphenols in an in vitro cell culture system. In addition, B-type procyanidins may have a common core structure that is necessary for interacting with a target molecule to induce cytotoxicity; however, further studies are needed.

## 4. Materials and Methods

### 4.1. Materials

FLs from black soybean seed coats were prepared according to the method of Ito et al. [4]. The FL extraction contained 6.22% catechins, 6.35% B2, 2.69% C1, and 1.25% A2. As a reference, we also determined the polyphenol concentration in this fraction using the vanillin-sulfuric acid method (77.10%). EC was purchased from TCI Chemicals (Tokyo, Japan). B2, C1, and A2 were purchased from Phytolab GmbH & Co.KG (Vestenbergsgreuth, Germany). BSA was purchased from Sigma-Aldrich (St. Louis, MO, USA).

### 4.2. Analysis of Fluorescence Quenching of BSA

Analysis of fluorescence quenching was performed as previously described [27]. A series of mixtures were prepared with FLs (EC, B2, C1, and A2) at various concentrations, and a fixed concentration of BSA (0.075 mg/mL). Following incubation of the mixture at 20 °C for 20 min, the fluorescence spectrum of BSA in the mixture was measured at 20 °C in 50 mM sodium phosphate buffer (pH 7.0) at an excitation wavelength of 280 nm and emission wavelength range of 290–650 nm. All measurements were performed in triplicate. Fluorescence quenching data were analyzed by the Stern–Volmer equation (Equation (1)):

F_0_ = F (1 + Kq × Q) (1)

where F_0_ and F are the relative fluorescence intensities of BSA without and with FLs or procyanidins, respectively, Kq is the Stern–Volmer quenching constant, and Q is the concentration of FLs or procyanidins.

### 4.3. Molecular Docking Experiment

The drug-bound BSA structure (PDB code: 4JK4, Resolution: 2.653 A) was obtained from the Protein Data Bank (PDB) [28]. The co-crystalized structure was prepared using MOE 2019.01 for correction of structural issues (such as broken bonds, missing loops, etc.), addition of hydrogens, and calculation of partial charges. The 2D structures of the FLs were downloaded from CS ChemDraw (ver.16.0.1.4, PerkinElmer Informatics, Inc., Waltham, MA, USA) in the MOL file format and converted to 3D structures in MOE via energy minimization. MOE-Docking was used for docking simulation of the FLs and prediction of the binding affinity with the BSA protein structure. The original drug-binding pocket was chosen as the active site for docking. Site Finder in MOE was also used to identify the potential binding pockets and analyze the conserved pocket residues. Classical triangle matching was chosen as the placement method, and the number of placement poses was set to 100. The output docking poses were evaluated by the London dG score and the top 30 poses were chosen. Next, the rigid receptor method was employed in the refinement step. The number of the final output docking poses was set to 20, followed by minimization using the Amber10: EHT force field in MOE. The GBVI/WSA dG score was used to estimate the free energy of binding of the FLs with BSA. The binding mode was analyzed in MOE after the refinement minimization [29,30].

### 4.4. Cytotoxicity Test

F11 cells, a mouse N18TGneuroblastoma X rat DRG sensory neuron hybrid cell line, were purchased from Sigma-Aldrich (St. Louis, MO, USA). The F11 cells were grown to near confluence in monolayer cultures at 37 °C in an atmosphere of 5% CO_2_ in Dulbecco′s modified Eagle′s medium (DMEM) containing 10% (*v*/*v*) FBS. 5.0 × 10^3^ cells were incubated for 24 h in 96-well culture plates containing DMEM with 10% FBS. The cells were washed with PBS and incubated in 2 different culture media: (1) control medium supplemented with 10% FBS, and (2) control medium supplemented with 2% synthetic serum XerumFree™ XF205 (XF; TNCBIO, Eindhoven, Netherlands) with various concentrations of FLs for 48 h. We confirmed that similar F11 cell growth was shown in both 10% FBS and 2% XF. After incubation, 1 μM Calcein-AM (3’,6’-di(*O*-acetyl)-4’,5’-bis [*N*,*N*-bis(carboxymethyl) aminomethyl] fluorescein tetraacetoxymethyl ester; DOJINDO, Tokyo, Japan) was added to each well and incubated for 30 min. The fluorescence intensity of each cell was measured using a multilabel plate reader, Arvo-X4 (Perkin Elmer, MA, USA), with an excitation filter: 485 nm, emission filter: 535 nm [31]. Cell viability was calculated as the ratio of the fluorescence intensity of the group with FLs added to the group without FLs added. The cytotoxicities of the other procyanidins in 2% XF were determined as described above.

### 4.5. Statistical Analysis

Data are expressed as means ± SD unless otherwise stated from at least triplicate independent analyses. Statistical analysis of the data was performed by Dunnett′s test or the Kruskal Wallis test followed by Mann Whitney tests. A probability of *p* < 0.05 was considered statistically significant.

## Figures and Tables

**Table 1:** Quenching constant (Kq) for interaction of EC, B2, C1 and A2 with BSA calculated using the Stern–Volmer equation.

	Kq (×10^−3^, L·mol^−1^·s^−1^)	Coefficient of Determination (R^2^)
(−)-epicatechin (EC)	7.44 ± 2.13 ^1^	0.0157
procyanidin B2 (B2)	16.35 ± 4.37	0.9201
procyandin C1 (C1)	14.33 ± 4.98	0.8957
cinnamtannin A2 (A2)	29.28 ± 7.47	0.9987

^1^ Each value represents mean ± S.D.

## Supplementary Materials

The following are available online. Figure S1: Emission spectra of BSA (**a**), FLs (**b**), EC (**c**), B2 (**d**), C1 (**d**) and A2 (**f**) measured at an emission wavelength of 350 nm.
