# Supplementary material for: Elucidation of the Interaction between Flavan-3-ols and Bovine Serum Albumin and Its Effect on Their In-Vitro Cytotoxicity
Source: Molecules. 2019 Oct 11;24(20):3667. doi: 10.3390/molecules24203667 (PMC6832702; doi:10.3390/molecules24203667)
Supplement: Supplementary file 1 [file molecules-24-03667-s001.pdf]

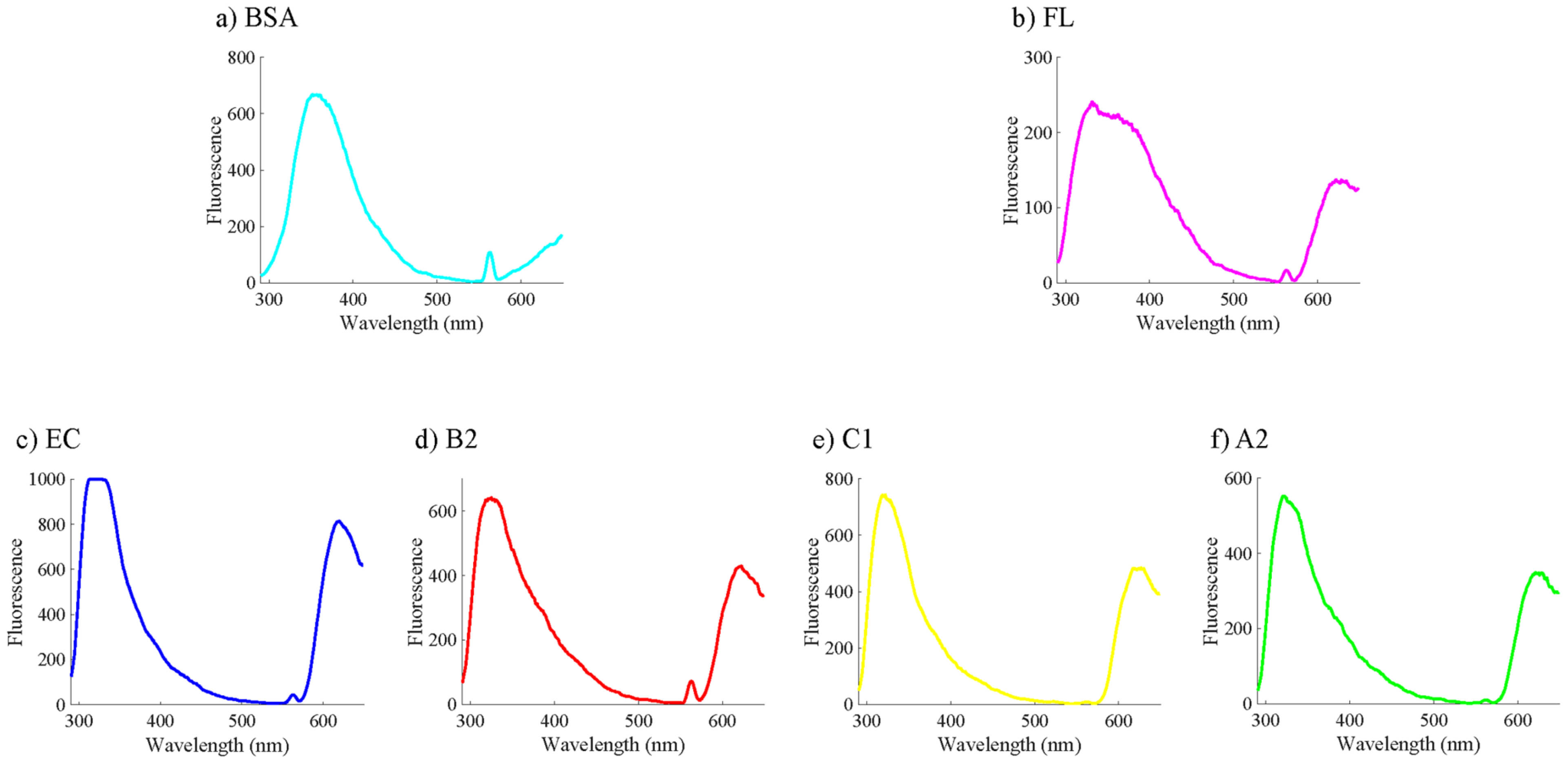

Figure S1 Emission spectra of BSA (a, 0.03mg/mL), FL (b, 62.5 μ g/ml), EC(c, 50 μ M), B2(d, 50 μ M), C1 (e, 50 μ M) and A2(f, 50 μ M)(λ EX ~ 280 nm, λ EM ~ 350 nm)
